# Leptomeningeal relapse in primary cutaneous DLBCL: Implications for a prophylactic CNS therapy

**DOI:** 10.1002/cnr2.1295

**Published:** 2020-10-07

**Authors:** Deepak Sundriyal, Lima Arya, Ruchi Srivastava, Meenu Walia, Amit Sehrawat

**Affiliations:** ^1^ Department of Medical Oncology, Hematology All India Institute of Medical Sciences Rishikesh India; ^2^ Department of Medical Oncology Max Superspeciality Hospital New Delhi India; ^3^ Department of Pathology Max Superspeciality Hospital New Delhi India

**Keywords:** leptomeningeal relapse, PCDLBCL leg type, prophylactic CNS therapy

## Abstract

**Background:**

Isolated leptomeningeal relapse in a case of cutaneous lymphoma is an uncommon event more so in a case of primary cutaneous diffuse large B‐cell lymphoma (PCDLBCL). This phenomenon is of great significance as the subsequent prognosis becomes poor and the prophylactic central nervous system (CNS) therapy if administered, can reduce the chances of relapse, however, the survival benefit remains uncertain. The role of prophylactic CNS therapy is not well defined in the case of PCDLBCL.

**Case:**

We report a case of PCDLBCL leg type with a low CNS International Prognostic Index (CNS‐IPI) risk, who developed isolated leptomeningeal relapse in the form of bilateral facial nerve palsy. He was managed by 2nd line chemotherapy and CNS directed therapy and achieved complete remission.

**Conclusion:**

PCDLBCL leg type is an aggressive malignancy. Molecular/genomic mechanism likely responsible for CNS dissemination should be identified by prospective multi‐centric studies that can better define the subsets of patients eligible for prophylactic therapy in the absence of a high CNS‐IPI risk.

## 1 INTRODUCTION

Primary cutaneous diffuse large B‐cell lymphoma (PCDLBCL) leg type is a rare extranodal non‐Hodgkin lymphoma. Approximately 4% of all cutaneous lymphomas are comprised of PCDLBCL, leg type. It is an aggressive malignancy primarily affecting elderly females in their sixth or seventh decades of life. Lymphomatous lesions are present in the skin and subcutaneous tissue in the lower legs with no evidence of extracutaneous involvement at the time of initial diagnosis, hence the name. However, other cutaneous sites may be involved in 10% of the patients. A combination of morphological, histological, immunohistochemical, and molecular markers is necessary for a definite diagnosis. The disease is rapidly progressive, and the outcomes are poor with the standard treatment protocols. We present a case of PCDLBCL leg type, who developed isolated leptomeningeal relapse during the course of treatment.

## 2 CASE

A 61‐year‐old male presented to us with a lump on his lower part of back of 4 weeks' duration. The lump was rapidly increasing in size with mild pain. He denied similar lesions elsewhere. On examination, there was an ill‐defined swelling in the lower part of the back, fixed to the skin and the underlying tissues. The Zubrod performance status (PS) was zero. An incisional biopsy of the lesion was done, which was suggestive of intermediate to large size round tumors cells arranged in sheets with interspersed histiocytes, infiltrating into the dermis and subcutaneous tissue suggestive of lymphoma. Immunohistochemistry (IHC) markers were ordered, and a positron emission tomography‐computed tomography (PET‐CT) scan was advised. PETCT was suggestive of a 6.8 × 4 cm cutaneous‐subcutaneous lesion with an SUV max of 22.5 situated at the lower back at the level of L4‐L5 vertebra. No other hypermetabolic focus was noted in the rest of the visualized body. On IHC, tumor cells were positive for CD 20, BCL2, BCL6, and MUM1 while being negative for CD3, MYC, and CD10. The Ki‐67 labeling index was 60%.

Serum lactate dehydrogenase (LDH) levels were 157 U/L (range). Blood counts including peripheral smear, tumor lysis panel, and liver function tests were normal. Viral marker panel for hepatitis B, hepatitis C, and HIV was negative. No atypical cells were seen on bone marrow examination. A final diagnosis of PCDLBCL, leg type stage IE, non‐germinal center B, CNS International Prognostic Index (CNS‐IPI) risk low, was reached.

The patient was planned for R‐CHOP protocol chemo‐immunotherapy. CNS prophylaxis was not given because of low CNS‐IPI risk. He received 4 cycles of chemotherapy without any untoward incident. A PET‐CT done on day 14 post fourth cycle, was suggestive of partial metabolic as well as a morphological response. Further, 2 cycles were advised.

**FIGURE 1:**
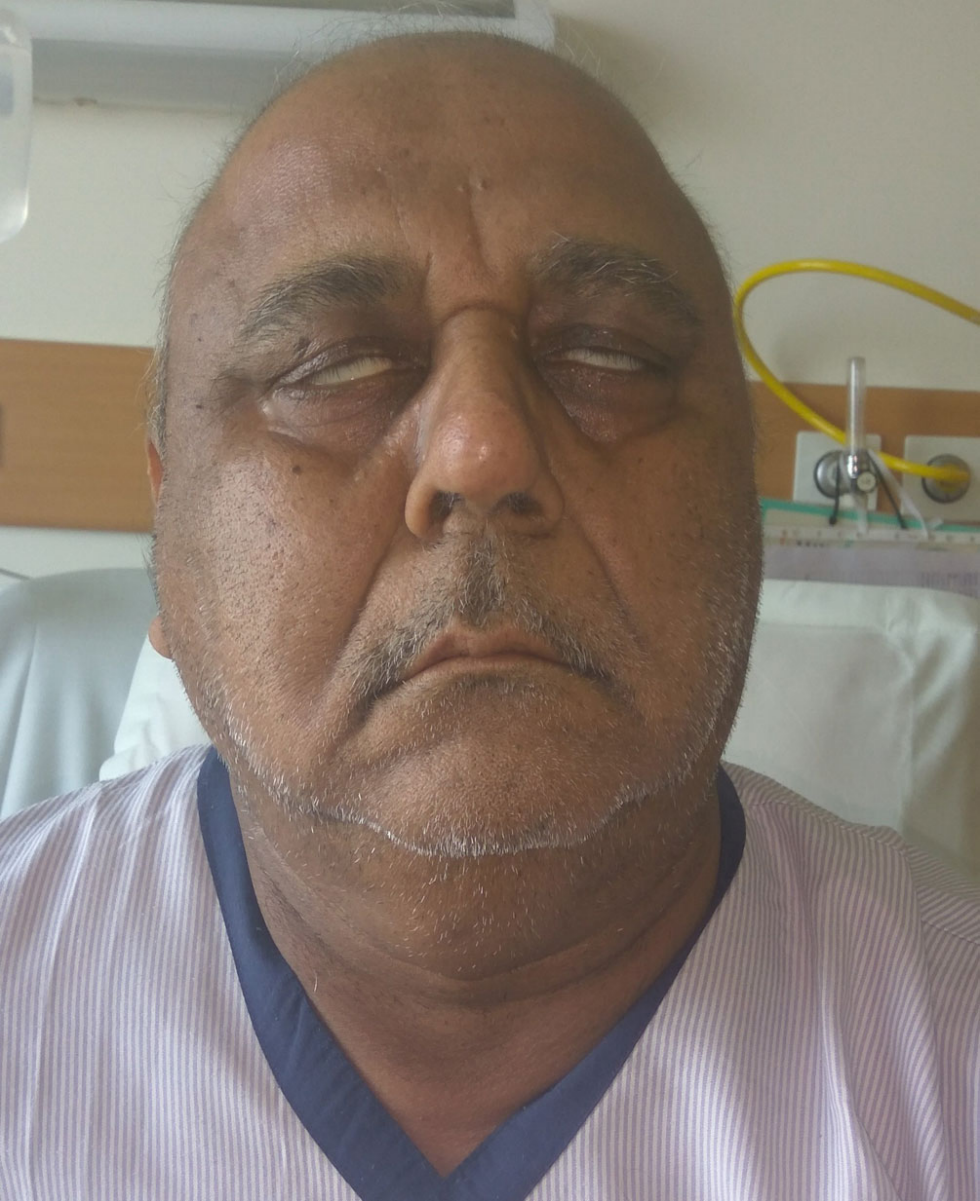
Bilateral Facial nerve palsy

He reported to us 2 days before the 5th cycle with a 3‐days history of headache, inability to close the eyes, and drooling of liquids from both angles of the mouth (Figure 1). On examination, he had bilateral lower motor neuron facial nerve palsy. Zubrod PS was preserved. The rest of the clinical examination was non‐contributory.

**FIGURE 2:**
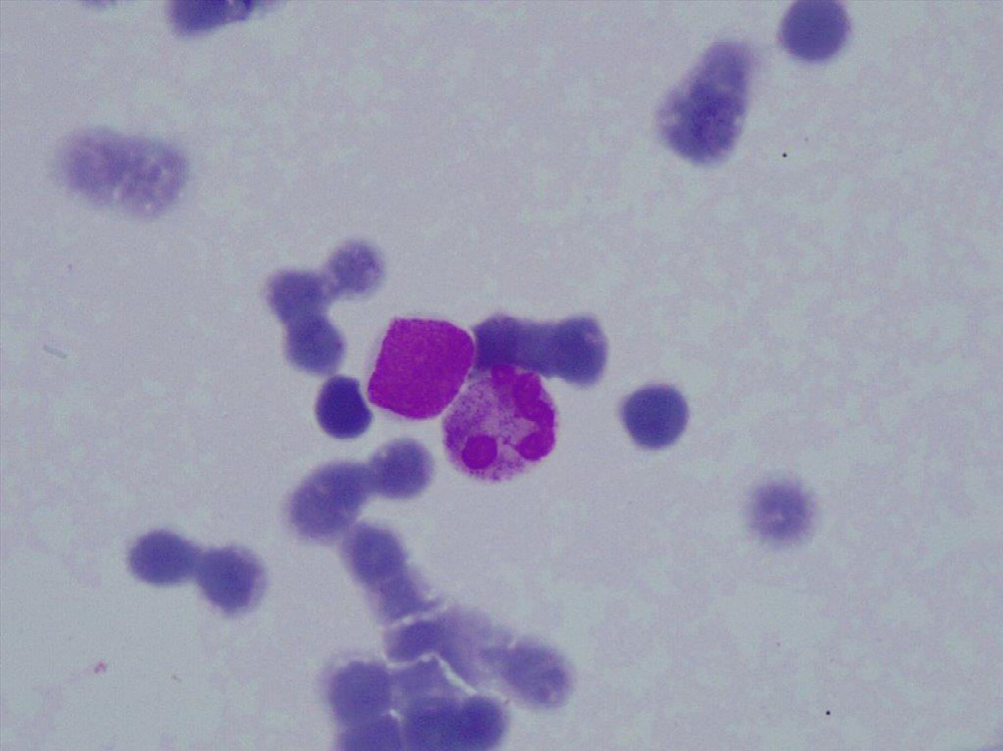
Cytocentrifuge preparation of cerebrospinal fluid examination (CSF) showing atypical lymphoid cell along with a neutrophil (May Grunwald Giemsa100X)

Contrast‐enhanced magnetic resonance imaging of the brain was negative for any intra‐cranial lesion and leptomeningeal enhancement. Complete blood counts, metabolic panel, liver function, kidney function, and coagulation studies were normal. He was admitted to the day‐care and a diagnostic lumbar puncture was done, and cerebrospinal fluid examination (CSF) was ordered. CSF examination revealed elevated proteins (139 mg/dL) and increased total cell count (140/μL) with a lymphocytic predominance. Infiltration by atypical lymphoid cells was seen, ruling a diagnosis of leptomeningeal metastasis from PCDLBCL, leg type (Figure 2).

## 3 OUTCOME

He was planned for a 2nd line chemotherapy (R‐GEMOX protocol) along with CNS‐directed therapy in the form of intrathecal (IT) triple regimen consisting of methotrexate 12 mg, cytarabine 30 mg, and hydrocortisone 50 mg twice weekly. Facial paralysis improved partially after the second week of the treatment. A repeat CSF examination at the beginning of the fourth week revealed normalization of protein levels and the absence of malignant cells. He achieved a complete metabolic remission after 4 cycles of chemotherapy on PET‐CT and intrathecal therapy. He was advised an autologous stem cell transplant; however, he refused it citing financial reasons and decided to continue the treatment at a nearby facility. He received 6 cycles of the same protocol along with the maintenance IT chemotherapy once a week, CSF showing biochemical and cytological remission.

## 4 DISCUSSION

Our case report highlights two important aspects related to DLBCL and CNS relapse.

First, isolated CNS relapse in a case of DLBCL is rarely seen in the era of rituximab based immune‐chemotherapy. The UK NCRI R‐CHOP‐14 vs 21 trial reported only 11 (1.01%) cases of isolated CNS relapse out of 1080 patients. Furthermore, isolated leptomeningeal involvement was reported in only three patients (0.27%) with the remaining involving the brain parenchyma.^1^ An analysis of patients treated with CHOP‐14 with or without rituximab in the RICOVER‐60 trial of the German High‐Grade Non‐Hodgkin Lymphoma Study Group (DSHNHL) revealed only 34/1222 patients (2.78%) developing isolated CNS relapse after achieving complete or partial remission. Leptomeningeal relapse was seen in only 15/1222 (1.22%) patients.^2^ Moreover, isolated bilateral facial nerve palsy heralding relapse of DLBCL is very rarely seen, and we came across only a few cases after an extensive literature search.^3^, ^4^

Second, the role of prophylactic CNS therapy in the case of PCDLBCL‐leg type, in the absence of a high CNS‐ IPI score, is controversial. The CNS‐IPI is a risk model consisting of the IPI factors (age >60 years, serum LDH > normal, performance status >1, stage III or IV, and extranodal involvement >1 site) in addition to the involvement of kidneys and/or adrenal glands. This is a highly reproducible model that can be used to estimate the risk of CNS relapse/relapse in patients treated with R‐CHOP chemotherapy. According to this model, 90% of the patients with DLBCL belong to the low (score of 0 and 1), and intermediate‐risk (score of 2 and 3) group and have a CNS relapse risk of <5%; they may be spared any diagnostic and therapeutic intervention. In contrast, those in the high risk (score of 4, 5, and 6) group have a >10% risk of CNS relapse and should be considered for CNS‐directed investigations and prophylactic interventions. Among the specific extranodal sites, only the kidney or adrenal gland involvement was significantly associated with CNS relapse or relapse. Skin involvement was not included in the analysis as only a few events were reported, and details were missing.^5^ An international multicentre study of 1532 patients treated with chemoimmunotherapy identified secondary CNS involvement in only 62(4%) patients. In this study, disease stage III/IV, elevated serum LDH, kidney/adrenal, and uterine/testicular involvement were independently associated with secondary CNS involvement.^6^ However, the Involvement of the skin significantly increased the risk of CNS disease in the German High‐Grader Non‐Hodgkin Lymphoma Study Group and the MAbThera International Trial (DSHNHL/MInT) data.^7^, ^8^

British Committee for the standards in hematology recommend prophylaxis for those with elevated LDH and >1 extranodal site, or with testicular, breast, or epidural involvement.^9^ Similarly, the Spanish Lymphoma Group recommends CNS prophylaxis for high CNS‐IPI, double‐hit lymphoma, and the involvement of the kidney or adrenal gland without mentioning cutaneous lymphoma.^10^ PCDLBCL leg type is an uncommon, aggressive malignancy, and CNS relapse has been a rare event. Gardette et al and Bekkenk et al identified CNS relapse only in 3.7% and 4.5% of the cases of PCDLBCL leg type, respectively. Patients with PCDLBCL leg type died more frequently due to the involvement of non‐CNS organ systems. CNS prophylaxis is hence, not recommended due to the paucity of data and rarity of CNS involvement.^11^, ^12^

## 5 CONCLUSION

PCDLBCL leg type is an aggressive malignancy. CNS relapse is a rare event and currently, it is debatable to recommend prophylactic CNS therapy in the absence of a high CNS‐IPI score. Since it is an uncommon malignancy, molecular/genomic mechanism likely responsible for CNS dissemination should be identified by prospective multi‐centric studies that can better define the subsets of patients eligible for prophylactic therapy as well as the survival benefit.

## CONFLICT OF INTEREST

The authors declare that they do not have any conflicts of interest.

## AUTHOR CONTRIBUTIONS

D.S. and L.A. collected and interpreted the data. D.S., A.S., and M.W. prepared the manuscript. A.S. and L.A. were involved in patient management. All authors read and approved the final manuscript.

## ETHICS STATEMENT

Approval was taken from the Institutional Ethics Committee and the patient for publication of this report.

## Data Availability

Data available on request from the authors.
